# Design of Novel Amphipathic α-Helical Antimicrobial Peptides with No Toxicity as Therapeutics against the Antibiotic-Resistant Gram-Negative Bacterial Pathogen, *Acinetobacter Baumannii*

**Published:** 2019-05-30

**Authors:** Colin T Mant, Ziqing Jiang, Lajos Gera, Tim Davis, Robert S Hodges

**Affiliations:** 1 Department of Biochemistry and Molecular Genetics, University of Colorado, School of Medicine, Anschutz Medical Campus, Aurora, Colorado, USA; 2 AMP Discovery LLC, Aurora, Colorado, USA

**Keywords:** Gram-negative pathogen, *Acinetobacter baumannii*, Amphipathic α-helical peptides, Antimicrobial peptides (AMPs), Specificity determinants, Hemolytic activity, Polar face positively charged residues (D-Lys, L-Dab and L-Dap)

## Abstract

We designed *de novo* and synthesized two series of five 26-residue amphipathic α-helical cationic antimicrobial peptides (AMPs) with five or six positively charged residues (D-Lys, L-Dab (2,4-diaminobutyric acid) or L-Dap (2,3-diaminopropionic acid)) on the polar face where all other residues are in the D-conformation. Hemolytic activity against human red blood cells was determined using the most stringent conditions for the hemolysis assay, 18h at 37°C, 1% human erythrocytes and peptide concentrations up to 1000 μg/mL (~380 μM). Antimicrobial activity was determined against 7 *Acinetobacter baumannii* strains, resistant to polymyxin B and colistin (antibiotics of last resort) to show the effect of positively charged residues in two different locations on the polar face (positions 3, 7, 11, 18, 22 and 26 *versus* positions 3, 7, 14, 15, 22 and 26). All 10 peptides had two D-Lys residues in the center of the non-polar face as “specificity determinants” at positions 13 and 16 which provide specificity for prokaryotic cells over eukaryotic cells. Specificity determinants also maintain excellent antimicrobial activity in the presence of human sera. This study shows that the location and type of positively charged residue (Dab and Dap) on the polar face are critical to obtain the best therapeutic indices.

## Introduction

The growing emergence of pathogenic bacteria with clinically significant resistance to conventional antibiotics is a major public health concern [1-5]. As noted by Falanga, et al. [6] we are facing a worldwide re-emergence of infectious diseases and a rapid increase in multidrug-resistant (MDR) bacteria, threatening the world with a return to the pre-antibiotic era. Indeed, there are now “Superbugs” that are resistant to most or all available antibiotics [7]. The scope of the challenge in tackling drug-resistant infections globally is reported in detail in a 2016 review on antimicrobial resistance [4]. Thus, it was estimated that, by 2050, 10 million lives a year will be at risk due to the rise of drug-resistant infections if proactive solutions are not quickly found to slow the rate of drug resistance. At present, 700,000 people die every year from drug resistant strains of common bacterial infections, HIV, TB and malaria [4]. While the 2016 review [4] offered a plethora of approaches to slowing down or preventing future bacterial resistance to antibiotics (e.g., promoting vaccine use, avoiding unnecessary antibiotic use, better water and sanitation, decrease in environmental pollution), the fact remains that organisms resistant to conventional antibiotics will still be present and must be dealt with. Indeed, it has frequently been asserted that, as part of a global response to MDR bacteria, we must increase the number of effective antimicrobial drugs to defeat infections that have become resistant to existing antibiotics [4]. Unfortunately, antibiotic discovery has stalled just as we need it the most. Between 1929 and the 1970s, more than 20 new classes (not just analogs of an existing class) of antibiotic reached the market [3]. Since then, only two new classes have reached the market, with the worldwide antibiotic pipeline for new antibiotic classes active against highly resistant Gram-negative bacteria being almost non-existent [3]. It is estimated that only 4 new classes of antibiotics can be expected in the next 30 years, while antibiotic resistance to some pathogens may more than double in the same period [4]. Although, in the 1970s and 1980s, the pharmaceutical industry did produce a stream of antibiotics, these were not new classes but analogs of existing classes [3]. The fundamental problem with this approach is that, although analog development is low risk compared to novel class discovery and development, analogs eventually became more difficult to come by and the process hits a dead end.

A potential solution to the crisis of medically resistant strains of bacteria lies in a ubiquitous response in nature to bacterial infections, namely the production of antimicrobial peptides (AMPs) [6,8-24]. AMPs are produced by a wide variety of organisms, including bacteria, fungi, plants, insects, amphibians, crustaceans, fish and mammals (including humans) [25]. AMPs (specifically, cationic AMPs) are fast-acting bactericides with generally broad spectrum activity [25]. In addition, AMPs in general do not have specific targets (unlike traditional antibiotics), their mode of action generally involving nonspecific interactions with the cytoplasmic membrane of bacteria. This causes peptide accumulation in the membrane, leading to increased permeability and loss of barrier function [8,9,12,13,15-30]. Development of resistance is not expected since this would require substantial changes in the lipid composition of the cell membranes of microorganisms. The majority of AMPs in current clinical development target skin infections caused by Gram-positive bacteria, i.e., topical use only [31]. In addition, within the last 30 years, only four natural AMPs have found their way onto the market and no systemic AMP has been approved by the Federal Drug Administration in the USA [31]. This dearth of clinically approved AMPs despite the past three decades of attempts and the excellent antimicrobial activity of many AMPs lies mainly in their generally high toxicity to normal cells which prevents their use as a systemic drug. Interestingly, cationic AMPs polymyxin B and polymyxin E (colistin) saw widespread use in the 1960s and 1970s. However, their clinical use in the 1970s was scaled back considerably due to serious neurotoxicity and nephrotoxicity issues [32-36]. Despite these toxicity drawbacks, these two peptides returned as antibiotics of last resort with the emergence of prevalent Gram-negative bacteria with multidrug resistance. However, the aforementioned emergence of polymyxin-resistant “Superbugs” [32,33,36], due to the fact that these particular peptide antibiotics (unlike the AMPs presently under consideration) have specific targets and are thus prone to resistance, means that it is now critical to develop antimicrobials effective against both polymyxin B- and colistin-resistant microorganisms. Worldwide research for the past 30 years to remove toxicity from AMPs, thus enabling a shift of focus from development of peptide drugs for topical use towards agents for systemic administration, has been unsuccessful until recent work in our laboratory.

Numerous structure/activity studies on both natural and synthetic AMPs identified factors important for antimicrobial activity: the presence of both hydrophobic and basic (positively charged) residues, an amphipathic nature, and preformed or inducible secondary structure (α-helix or β-sheet) [16]. We have always postulated that a *de novo* design synthetic peptide approach to examining the effect of incremental changes in these parameters would enable rapid progress in the rational design of novel peptide AMPs. Thus, from lessons learned about factors important for antimicrobial activity, as noted above, we utilized the structural framework, or template, of a 26-residue amphipathic α-helical AMP with excellent antimicrobial activity but with, initially, strong hemolytic activity [16]. The 26-residue length of the template was designed to be able to accept amino acid substitutions with minimal effects on peptide properties and stability other than the ones under investigation; at the same time, synthesis and purification of analogs remained straightforward. With this template approach, we determined the effect on biological activity of varying the hydrophobicity of the non-polar face [37] or the number of positively charged residues on the polar face [38]. In addition, utilizing D-enantiomers of amino acids led to excellent stability against proteolytic digestion (a key property for AMPs to be useful as injectable AMPs), whilst maintaining excellent antimicrobial activity [39].

At this point, a major milestone was our discovery of “specificity determinants” allowing selectivity between eukaryotic cells and Gram-negative microorganisms, i.e., producing a major decrease in toxicity as measured by hemolysis of human red blood cells [39-41]. These “specificity determinants” were one, later two, Lys-substitutions in the middle of the non-polar face of the amphipathic model peptide, a peptide long enough to allow such substitutions whilst maintaining sufficient hydrophobicity on the non-polar face. Briefly, we utilized positively charged residues as specificity determinants (Lys residues at positions 13 and 16 of the non-polar face) of the 26-residue peptide. In addition, we manipulated total hydrophobicity, hydrophobe type and location as design parameters. Taken together, these approaches resulted in unprecedented, at that time, improvements in therapeutic indices (hemolytic activity/antimicrobial activity) [40,41]. This discovery hastened another aspect of our template design, namely the requirement for our peptides to lie parallel to the membrane, surface, i.e., promoting the “carpet model” of interaction [24,42,43] while preventing penetration of the membrane as a transmembrane helix in eukaryotic cells *via* a “barrel stave” mechanism [24,44], thus preventing hemolysis. Further, we demonstrated that modification of native AMPs (the 22-residue Piscidin 1 and 28-residue Dermaseptin S4) with Lys specificity determinants in the non-polar face of these amphipathic α-helical peptides produced similar results of improved antimicrobial activity and dramatically decreased hemolytic activity [45,46]. Such results are of critical importance to the future of AMPs as therapeutic agents.

With our focus now on developing a better Gram-negative AMP rather than to maintain broad-spectrum activity in a “one size fits all” approach, thus hastening development of such AMPs, we recently turned our attention to the polar face of our peptide template [47]. Namely, we replaced the Lys residues with Arg residues and unusual amino acids: ornithine (Orn) [22,48-53], diaminobutyric acid (Dab) [22,48-53] or diaminopropionic acid (Dap) [22,48-54]. Excitingly, AMPs with specificity determinants and with L-Dab and L-Dap on the polar face have essentially no hemolytic activity at high peptide concentrations (1000 μg/mL), demonstrating for the first time the importance of these unusual amino acid residues in solving long-standing hemolysis issues of AMPs, whilst maintaining excellent antimicrobial activity against seven *Acinetobacter baumannii* strains, resistant to polymyxin B and colistin, and 20 *A. baumannii* isolates from 2016 and 2017 with resistance to 18 different antibiotics.

The present study serves to continue the success of our template-driven *de novo* design approach by attempting to fine-tune our recent achievement of utilizing unusual amino acids (Dab and Dap) in the polar face of our AMP to eliminate hemolysis [47]. Thus, we have determined the effect of changing locations of positively charged residues on the polar face of the AMP, as well as eliminated a single positively charged residue at the C-terminal which allows future development of Pegylated AMPs on a C-terminal cysteine residue if prolonged half-life is necessary.

## Materials and Methods

### Solid-phase peptide synthesis and reversed-phase purification

The synthesis and purification methods have been described in detail in a previous publication [47].

### Characterization of helical structure

Circular dichroism (CD) spectroscopy was used to determine the mean residue molar ellipticities of the peptides, using a Jasco J-815 spectropolarimeter (Jasco, Inc., Easton, MD, USA) under two sets of conditions: at pH 7.0 the buffer was 50 mM NaH_2_PO_4_/NaHPO_4_/100 mM KCl and in the presence of an α-helix inducing solvent, 2, 2, 2-trifluoroethanol, TFE, (50 mM NaH_2_PO_4_/NaHPO_4_/100 mM KCl, pH 7.0 buffer/50% TFE). A 10-fold dilution of an approximately 500μM stock solution of the peptides was loaded into a 0.1 cm quartz cell and its ellipticity scanned from 195 to 250 nm. Peptide concentrations were determined by quantitative amino acid analysis.

### Amino acid analysis for peptide quantitation

The method of Cohen and Michaud [55] was used for amino acid analysis. Each peptide sample was aliquoted into glass tubes and lyophilized followed by acid hydrolysis in 6 M HCl with 0.1% phenol for 48 h at 110°C. The resulting solution was allowed to come to room temperature and then vacuum-dried to remove the HCl. Each sample was then resuspended in 10 mM HCl and 20 μL of sample was added to 60 μL of 0.2M sodium borate buffer, pH 8.8. To this mixture, 20 μL of 6-aminoquinoyl-N-hydroxysuccinimidyl carbamate in acetonitrile was added to derivatize the amino acids present in the sample. This sample was then heated to 55°C for 15 min to convert Tyr byproducts to one form. An Agilent 1260 series instrument with a Waters AccQTag column, 3.9 mm I.D. × 150 mm column was used to separate and quantify the derivatized amino acids present in each sample using UV absorbance at 254 nm.

### Gram-negative bacterial strains used in this study

The *A. baumannii* strains used in this study consisted of seven strains resistant to Polymyxin B and Colistin (antibiotics of last resort) obtained from MERCK (M89941, M89949, M89951, M89952, M89953, M89955 and M89963). The MIC_GM_ in this case is the geometric mean MIC from the seven *Acinetobacter baumannii* strains used in this study.

### Antimicrobial activity (MIC) determination

The minimal inhibitory concentration (MIC) is defined as the lowest peptide concentration that inhibited bacterial growth. MICs were measured by a standard microtiter dilution method in Mueller Hinton (MH) medium. Briefly, cells were grown overnight at 37°C in MH broth and were diluted in the same medium. Serial dilutions of the peptides were added to the microtiter plates in a volume of 50 μL, followed by the addition of 50 μL of bacteria to give a final inoculum of 5 × 10^5^ colony-forming units (CFU)/mL. The plates were incubated at 37°C for 24h, and the MICs were determined. The MIC_GM_ is the geometric mean of the number of MIC values.

### Hemolytic activity (HC_50_) determination

Peptide samples (concentrations determined by amino acid analysis) were added to 1% human erythrocytes in phosphate-buffered saline (100 mM NaCl, 80 mM Na_2_HPO_4_, 20 mM NaH_2_PO_4_, pH 7.4) and the reaction mixtures were incubated at 37°C for 18h in microtiter plates. Two-fold serial dilutions of the peptide samples were carried out. This determination was made by withdrawing aliquots from the hemolysis assays and removing unlysed erythrocytes by centrifugation (800 × *g*). Hemoglobin release was determined spectrophotometrically at 570 nm. The control for 100% hemolysis was a sample of erythrocytes treated with water. The control for no release of hemoglobin was a sample of 1% erythrocytes without any peptide added. Since erythrocytes were in an isotonic medium, no detectable release (<1% of that released upon complete hemolysis) of hemoglobin was observed from this control during the course of the assay. The hemolytic activity HC_50_ is the peptide concentration that causes 50% hemolysis of erythrocytes after 18h. HC_50_ was determined from a plot of percent lysis *versus* peptide concentration (μM) using 12 different concentrations up to 1000 micrograms per ml for 18h at 37°C. The average of 3 replicates is used with an average variance of less than 4%. Fresh human blood was obtained from Vitalant, Denver, CO, USA.

### Therapeutic index (T.I.) determination

The therapeutic index is a widely accepted parameter to represent the specificity of antimicrobial peptides for prokaryotic *versus* eukaryotic cells. It is calculated by the ratio of hemolytic activity (HC_50_) and antimicrobial activity (MIC_GM_). The MIC_GM_ in this case is the geometric mean MIC from the seven *Acinetobacter baumannii* strains used in this study. Thus, larger values of therapeutic index indicate greater specificity for prokaryotic cells. Thus, the therapeutic index is the HC_50_/MIC_GM_ ratio.

## Results and Discussion

### Peptide design, location and type of positively charged residue on the polar face

**Figure 1:**
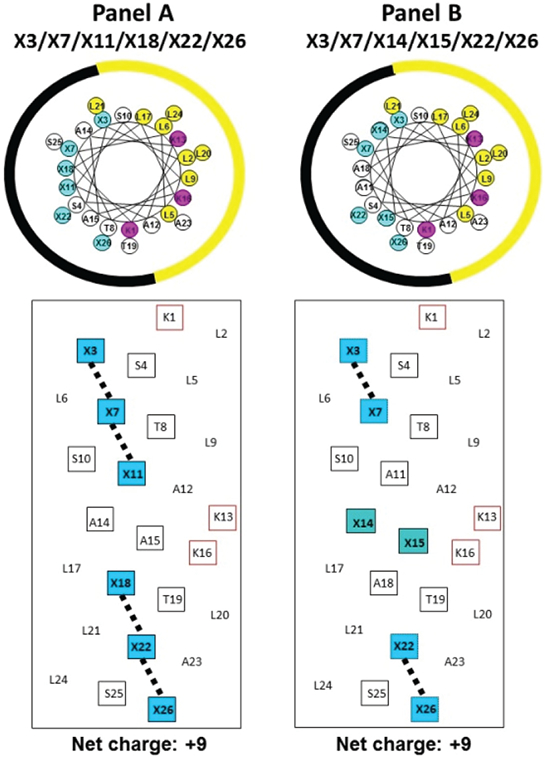
Helical wheels (upper panels) and helical nets (lower panels) representations of our helical AMPs. In the helical wheels, the non-polar face is indicated as a yellow arc (Leu residues are colored yellow and position Lys 1 and the specificity determinants at positions 13 and 16 are colored pink). The polar face is indicated as a black arc (positively charged residues are denoted by X and are colored blue).

In this study, we designed *de novo*, synthesized, purified and characterized ten potentially amphipathic α-helical antimicrobial peptides (AMPs) where we changed the location of positively charged residues on the polar face of the α-helix. Location 1 consists of five or six positively charged residues at positions 3, 7, 11, 18, 22 and 26 or 3, 7, 11, 18 and 22 (Figure 1). Location 2 has these residues at positions 3, 7, 14, 15, 22 and 26 or 3, 7, 14, 15 and 22 (Figure 1). The C-terminal positively charged residue (position 26) was replaced from both sets of AMPs with Ser 26 (Table 1). All ten AMPs have two “specificity determinants” (D-Lys residues at 13 and 16 in the center of the non-polar face). We have previously shown the critical importance of “specificity determinants” in these AMPs which encoded selectivity for Gram-negative pathogens and removed both Gram-positive activity and hemolytic activity from broad spectrum AMPs [40,41,45-47]. In addition, we have shown that specificity determinants have another important role of preventing high-affinity to human serum proteins [40,41,45,46].

**Figure 2:**
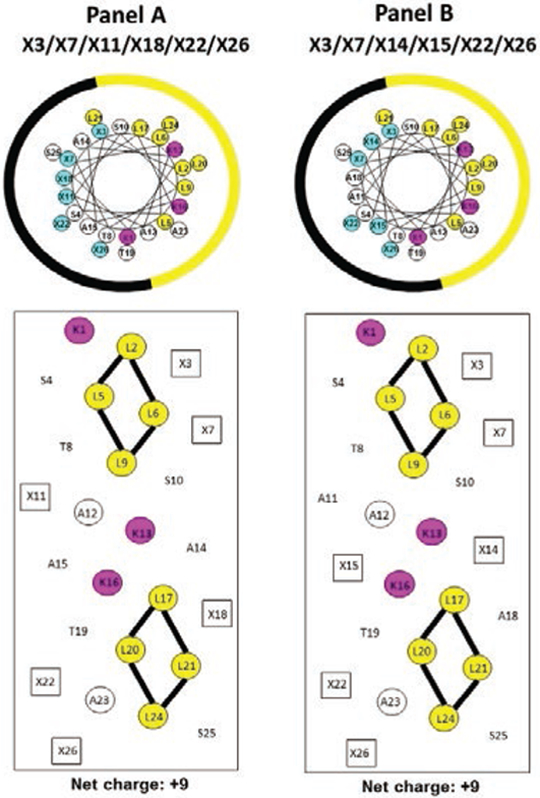
Helical wheels (upper panels) and helical nets (lower panels) representations of our helical AMPs. In the helical nets, the positively charged residues on the polar face are boxed and colored blue and other polar face residues are indicated with an open black box. The red open boxes denote Lys residues on the non-polar face (Lys 1 and specificity determinants Lys 13 and Lys 16). The positions denoted by X are the positions of positively charged residues on the polar face at positions 3, 7, 11, 18, 22 and 26 (Panel A) or at positions 3, 7, 14, 15, 22 and 26 (Panel B). The potential i to i +3 or i to i +4 electrostatic repulsions between positively charged residues are shown as black dotted lines.

Figure 1 shows a general amino acid sequence in a helical wheel and helical net representations where X denotes the positions on the polar face of the positively charged residues (colored blue). We have displayed two versions of the helical nets where the polar residues are displayed along the center of the helical net. Panel A shows the polar face residues at positions 3, 7, 11, 18, 22 and 26 and Panel B shows the polar face residues at positions 3, 7, 14, 15, 22 and 26. The major difference between the two relates to positions 11 and 18 in Panel A and positions 14 and 15 in Panel B. When the positively charged residues are at positions 3, 7, 11, 18, 22 and 26, we have denoted this orientation as −1 at the end of the peptide name (Table 1). When the positively charged residues are at positions 3, 7, 14, 15, 22 and 26, we have denoted this orientation as −2 at the end of the peptide name (Table 1). The −2 orientation creates a positively charged cluster in the sequence at positions 13, 14, 15 and 16. Positions 13 and 16 are the D-Lys residues as the specificity determinants on the non-polar face and positions 14 and 15 are on the polar face (D-Lys, L-Dab or L-Dap) residues. One of the most interesting points of this study was the substitutions of Dab and Dap residues in the L-conformation into an otherwise all D-antimicrobial peptide. The substitution of 5 or 6 positively charged residues on the polar face as either D-Lys, L-Dab or L-Dap (Table 1) was not expected to have any undesired effect on the conformation since our objective was to have as little α-helical structure as possible in aqueous conditions but maximum inducible α-helical structure in the presence of the hydrophobicity of the membrane (mimicked here by determining the helical structure by circular dichroism spectroscopy (CD) in the presence of 50% trifluoroethanol). We did not expect the 5 or 6 L-substitutions of Dab or Dap residues to affect the overall structure in any significant way since there are 20 or 21 positions out of 26 to maintain the structure in the presence of the hydrophobicity of the membrane. The use of the L-conformation for Dab and Dap residues was based on the fact that they are significantly less expensive for peptide synthesis and would not introduce any susceptibility to proteases since the Dab and Dap residues are unusual amino acids and are not recognized by proteases. The hydrophobic/non-polar faces of all ten AMPs have eight Leu residues in two clusters of four (colored yellow) separated by two Lys residues (specificity determinants in the center of the non-polar face (colored red)) (Figure 2). Position 1 in all peptides is D-Lys which we consider is on the non-polar face; thus, the non-polar face contains three D-Lys residues at positions 1, 13 and 16 to give a net charge of +3 on the nonpolar face and +5 or +6 on the polar face resulting in an overall net charge on these AMPs of either +9 or +8 depending on whether there are 6 or 5 positively charged residues on the polar face (Table 1).

In the helical wheels, the non-polar face is indicated as a yellow arc (Leu residues are colored yellow and position Lys 1 and the specificity determinants at positions 13 and 16 are colored pink). The polar face is indicated as a black arc (positively charged residues are colored blue). In the helical nets, the residues on the non-polar face are circled with the Lys residues colored red (Lys 1 and the specificity determinants, Lys 13 and Lys 16) and the Leu residues in two clusters (L2, L5, L6, L9 for the N-terminal cluster and L17, L20, L21 and L24 for the C-terminal cluster). The black open boxes denote the positively charged residues on the polar face at positions 3, 7, 11, 18, 22 and 26 (Panel A) and positions 3, 7, 14, 15, 22 and 26 (Panel B). The potential i to i +3 or i to i + 4 hydrophobic interactions between large hydrophobes are shown as black solid lines.

### Antibacterial activity

Table 2 shows the antibacterial activities against 7 different *Acinetobacter baumannii* strains resistant to polymyxin B and colistin (antibiotics of last resort). The geometric mean MIC values were determined for the ten AMPs, where the positively charged residue was varied from D-Lys, L-Dab and L-Dap on the polar face in two different locations designated −1 or −2 (Table 2). The MIC_GM_ values for Lys residues on polar face at positions 3, 7, 11, 18, 22 and 26 was 0.5 μM and for positions 3, 7, 14, 15, 22 and 26 was 0.4 μM. This suggests that antibacterial activity is not significantly affected by the change in location of the positively charged residues on the polar face. Similarly, replacing D-Lys residues with L-Dab residues had very little effect on the MIC_GM_ (0.9 μM at positions denoted −1 and 0.7 μM at positions denoted −2). Replacing D-Lys residues with L-Dap residues was more significant (0.5 μM for D-Lys at positions −1 and 1.2 μM for L-Dap residues at positions −1). At the −2 location, D-Lys residues had a MIC_GM_ value of 0.4 μM, while L-Dap residues at these same positions resulted in a value of 0.8 μM. Clearly, shortening the side-chain from Lys to Dab and then to Dap has a small but significant effect on antibacterial activity. The MIC_GM_ changed from 0.5 μM to 0.9 μM to 1.2 μM for D-Lys, L-Dab and L-Dap, respectively, when in the −1 location (compare D84, D86 and D105, Table 2). The MIC_GM_ changed from 0.4 μM to 0.7 μM to 0.8 μM for D-Lys, L-Dab and L-Dap, respectively, when in the −2 location (compare D88, D89 and D106, Table 2). The largest difference in the two locations occurs when Dap residues are used (compare D105(Lys^1^-6 Dap-1) MIC_GM_ of 1.2 μM with D106(Lys^1^-6 Dap-2) MIC_GM_ of 0.8 μM). There seems to be a major advantage to have Dap residues at position 14 and 15 on the polar face rather than positions 11 and 18. Positions 14 and 15 are between the two specificity determinants (D-Lys residues) on the non-polar face at positions 13 and 16. This is creating a positively charged cluster in the sequence (D-Lys13, L-Dap14, L-Dap15 and D-Lys 16) (Table 2). In the big picture, the changes in the geometric mean MIC value are minor compared to the effect observed in hemolytic activity by changing the residues on the polar face from D-Lys to L-Dab and L-Dap residues (4 carbon atoms, 2 carbon atoms and 1 carbon atom in the side-chain, respectively (Table 2). We discovered that we can eliminate the positively charged residue at position 26 with no significant effect on the geometric mean MIC value (compare D86(Lys^1^-6 Dab-1), MIC_GM_ 0.9 μM to D102(Lys^1^-5 Dab-1), MIC_GM_ 0.7 μM and D89(Lys^1^-6 Dab-2), MIC_GM_ 0.7 μM to D104(Lys^1^-5 Dab-2), MIC_GM_ 0.8 μM) (Table 2).

### Hemolytic activity and therapeutic indices

**Figure 3:**
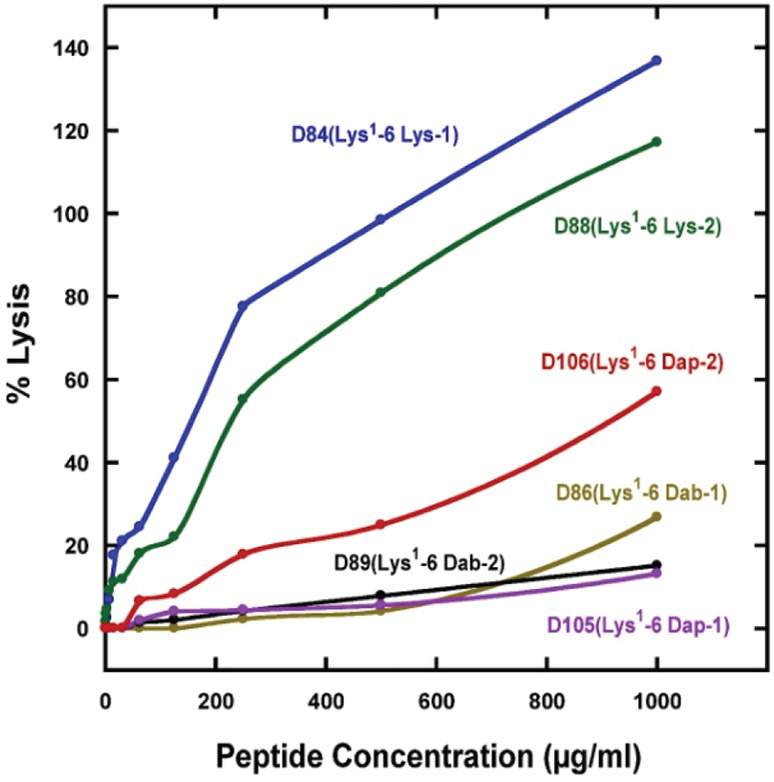
Percent lysis of human red blood cells *versus* peptide concentration of AMPs. The sequences of the six peptides (all containing Lys specificity determinants at positions 13 and 16 on the non-polar face and Lys 1 on the non-polar face are shown in table 1. Nomenclature, see table 1; 6 Lys-1 denotes six Lys residues on the polar face at positions 3, 7, 11, 18, 22 and 26; similarly, 6 Lys-2 denotes six Lys residues on the polar face at positions 3, 7, 14, 15, 22 and 26.

The biological activities of the ten peptide analogs are shown in table 2. The hemolytic activity is expressed as the HC_50_ value which is the concentration of peptide that results in 50% hemolysis of human red blood cells. In order to determine that we were able to eliminate hemolysis of human red blood cells, we used the most rigorous test of hemolytic activity (18h at 37°C and up to 1000 μg/mL or >350 μM of AMP). This is in stark contrast to other researchers who routinely use incubation times of just 0.5-2h. We have shown that, when the exposure time is increased from less than 2 h to 18h [16,20,39], substantially greater hemolysis is observed. Clearly, hemolysis should be monitored on human red blood cells for an exposure time up to 18h, since anything less will lead to misleading results. From figure 3, which shows the effect of peptide concentration on human red blood cells lysis, the decrease in hemolytic activity resulting from the use of the two unusual amino acid residues Dab and Dap on the polar face is dramatic. The Lys containing peptides (D84(Lys^1^-6 Lys-1) and D88(Lys^1^-6 Lys-2) have HC_50_ values of 54.3 and 80.6 μM and show 100% hemolysis at high peptide concentration (Figure 3 and Table 2). On the other hand, the two peptides containing Dab and Dap residues show essentially no lysis of human red blood cells at 1000 μg/mL as indicated by the linear lines (Figure 3) (D105(Lys^1^-6 Dap-1) and D89(Lys^1^-6 Dab-2)). The location of the positively charged residues on the polar face has a major effect on hemolysis and the best location is dependent on whether Dab or Dap residues are used. Using Dap residues in the −2 location (Dap at positions 14 and 15) results in significant more lysis of human red blood cells *versus* the −1 location (Dap at positions 11 and 18) compare D106(Lys^1^-6 Dap-2) to D105(Lys^1^-6 Dap-1 (Figure 3). See figure 1 to observe the difference in location on the polar face between −1 and −2 locations. When using Dab residues, the exact opposite effect is observed. Dab residues in the −2 location (Dab at positions 14 and 15) results in no measurable hemolysis at 1000 μg/mL (D89(Lys^1^-6 Dab-2), Figure 3). These results suggest that side-chain length, number of carbon atoms and location can affect hemolysis. The HC_50_ value is estimated when 50% hemolysis is not observed at 1000 μg/mL by extrapolation of the plots observed in figure 3. The therapeutic indices are calculated from the HC_50_ (μM)/MIC_GM_ (μM). We have also shown that we can remove the C-terminal positively charged residue and replace it with Ser26 without any consequence (Table 2). Compare removing the C-terminal Lys residue D84(Lys1-6 Lys-1) (TI=108.6) to D101(Lys1-5 Lys-1) (TI=129.9) and D88(Lys^1^-6 Lys-2) (TI=201.5) to D013(Lys^1^-5 Lys-2) (TI=192.7). Similarly, on removing the C-terminal Dab residue, compare D86(Lys^1^-6 Dab-1) (TI > 824) to D102(Lys^1^-5 Dab-1) (TI= >1012) and D89(Lys^1^-6 Dab-2) (TI> 1589) and D104(Lys^1^-5 Dab-2) (TI= >1863).

### Peptide hydrophobicity

**Figure 4:**
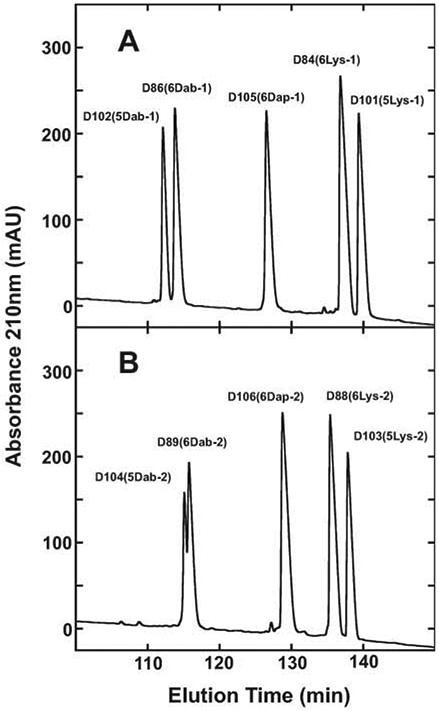
Relative hydrophobicity of AMPs as expressed by RP-HPLC elution time. Column: Zorbax 300 XB-C8, 150 × 2.1 mm ID, 5-μm particle size, 300-Å pore size; conditions, linear AB gradient (0.25% acetonitrile/min) at a flow-rate of 0.3 mL/min, where eluent A was 20 mM aq. TFA and eluent B was 20 mM TFA in acetonitrile and the temperature was 25°C. The sequences of the ten peptides are shown in table 1. Panel A represents peptides with substitutions of 6 positively charged residues on the polar face at positions 3, 7, 11, 18, 22 and 26 or 5 positively charged residues on the polar face at positions 3, 7, 11, 18 and 22 (−1 series; Table 1). Panel B represents peptides with 6 positively charged residues on the polar face at positions 3, 7, 14, 15, 22 and 26 or 5 positively charged residues on the polar face at positions 3, 7, 14, 15, and 22 (−2 series; Table 1).

Retention behavior in reversed-phase high-performance liquid chromatography (RP-HPLC) is an excellent method to represent overall peptide hydrophobicity. Even though the non-polar face of an amphipathic α-helical peptide represents the preferred binding domain for its interaction with the hydrophobic matrix of the reversed-phase column [56,57]; the overall hydrophobicity is also affected by the composition of residues on the polar face (five or six positively charged residues) (Figure 1). The RP-HPLC results for these two series of peptides are shown in figure 4 and table 3. Panel A shows the separation of five peptides with positively charged residues in the −1 location (positions 3, 7, 11, 18, 22 and 26 or 3, 7, 11, 18 and 22). Panel B shows the separation of the five peptides with positively charged residues in the −2 location (positions 3, 7, 14, 15, 22 and 26 or 3, 7, 14, 15, and 22). The type of positively charged residue on the polar face has a dramatic effect on the overall hydrophobicity, with the Dab residue being more hydrophilic (less hydrophobic) than the Dap residue even though the Dab residues are a carbon atom larger in their side-chain compared to the Dap residue: Dab peptide D86 (6Dab-1) retention time of 113.9 min compared to Dap peptide D105 (6Dap-1) retention time of 126.6.min (Panel A). Similarly, with these peptides in the −2 location, the D89 (6Dab-2) retention time was 115.8 min compared to the D106 (6Dap-2) peptide retention time of 128.8 min (Panel B). This can be explained by the Dab residues stabilizing the α-helical structure considerably more than the Dap residues. This means the polar face of the Dab peptides are interacting more with the hydrophobic matrix than the polar face of the Dap peptides, which results in a large decrease in retention time (t_R_ for Dap peptides is 126.6 min in the −1 location and t_R_ for Dab peptide is 113.9 min, i.e., a decrease of 12.7 min) or t_R_ for Dap peptides is 128.8 min in the −2 location and t_R_ for Dab peptide is 115.8 min, i.e., a decrease of 13.0 min). Compare Panel A and Panel B of figure 4 and table 3. All the +9 peptides shown in table 1 are identical in sequence except for the six polar face substitutions which are either D-Lys, L-Dab or L-Dap residues. Similarly the +8 peptides in table 1 have either 5 Lys or 5 Dab residues in two different locations (−1 or −2). The Lys peptides are always considerably more hydrophobic than the Dab peptides in location −1 or −2. This agrees with Lys peptides containing 4 carbon atoms in their side-chains relative to Dab peptides with 2 carbon atoms in their side-chains.

### Peptide helicity

The biophysical data for our ten peptides are shown in table 3. Circular dichroism (CD) spectroscopy was used to determine the α-helical content in aqueous conditions at pH 7 (50 mM PO_4_, 100 mM KCl) and in the presence of 50% trifluoroethanol (TFE) to mimic the hydrophobicity and α-helix inducing ability of the hydrophobic membrane, the target of our AMPs. Our strategy was to minimize α-helical structure in aqueous conditions and maximize the inducible α-helical structure in the presence of the hydrophobicity of the membrane. The % helix in aqueous conditions varied from 6 to 29% and the % inducible α-helix varied from 71 to 94% depending on the peptide (Table 3). The specificity determinants (Lys residues at positions 13 and 16 in the center of the non-polar face) were used to disrupt the continuous hydrophobic face of our template, creating two hydrophobic clusters of leucine residues, cluster one consisted of leucine residues at positions 2, 5, 6 and 9 and cluster two consisted of leucine residues at positions 17, 20, 21 and 24 (Figure 2). Though all AMPs met the general requirement of low α-helical content in aqueous conditions and dramatic increases in α-helical content in the presence of 50% TFE, there was no direct correlation with the type of positively charged residue (Lys, Dab or Dap) used on the polar face and helical content. In summary, inducible α-helical structure plays a critical role in providing our AMPs with desired properties.

## Conclusion

The goal of the present study was to determine whether our template-driven *de novo* designed peptide approach which enabled us to fulfill the long-sought goal of eliminating toxicity from AMPs could be further refined to improve therapeutic indices even more, as well as allow pegylation of the peptide model to enhance AMP half-life during therapeutic use, if required. Thus, our original 26-residue amphipathic α-helical AMP template, containing two D-Lys specificity determinants at positions 13 and 16 of the non-polar face and positively charged residues (D-Lys, L-Dab or L-Dap) at positions 3, 7, 11, 18, 22 and 26 of the polar face (Figures 1 and 2) were modified in two ways: (1) changing the positions of positively charged residues on the polar face originally at positions 11 and 18 (−1 orientation) to positions 14 and 15 (−2 orientation), the latter creating a positively charged cluster at positions 13, 14, 15 and 16 (Figures 1 and 2); and (2) eliminating the positively charged residue at position 26 through replacement with serine. Interestingly, the location of positively charged residues on the polar face had a major effect on hemolysis and the best location was dependent on whether Dab or Dap residues were used, i.e., side-chain length, number of carbon atoms and residue location all appear to affect hemolysis. Significantly, the therapeutic index of the 6Dab-containing −1 analog (>1012) rose to >1589 for the 6Dab-2 analog, an impressive increase in efficacy. In addition, the 5Dab-1 analog with a T.I. of >1012 saw an even greater increase in T.I. (>1863) for the 5Dab-2 peptide.

Comparing the 6Dab and 5Dab peptide series, the T.I. values for 6Dab-1 and 5Dab-1 were >824 and >1012, respectively; for the 6Dab-2 and 5Dab-2 peptides, the T.I. values were >1589 and >1863, respectively. Thus, we have shown that we can remove the C-terminal positively charged residue and replace it with Ser26 without any consequence; indeed, for the Dab analogs, an improvement in T.I. was observed. Such results now allow us to investigate the effectiveness of pegylation to a C-terminal Cys residue, in place of a positively charged residue, in order to prolong peptide half-life when desired.

Our continuing studies clearly show the potential of our amphipathic AMPs as potential therapeutics to replace existing antibiotics as well as the leading edge peptide design which our *de novo* designed template represents.

## Figures and Tables

**Table 1:** Polar face substitutions of positively charged residues in AMPs.

Peptide Name^a^	Net Charge	Sequence^b^												
		Specificity determinants (Lys13/Lys16) on non-polar face												
		1	3		7		11		18		22		26	
		KL	**X**	SLL	**X**	TLS	**X**	A**K**AA**K**L	**X**	TLL	**X**	ALS	**X**	
**D84 (Lys^1^-6 Lys-1)**	**+9**	Ac-KL	**(Lys)**	SLL	**(Lys)**	TLS	**(Lys)**	A**K**AA**K**L	**(Lys)**	TLL	**(Lys)**	ALS	**(Lys)**	-amide
**D86 (Lys^1^-6 Dab-1)**	**+9**	Ac-KL	**(L-Dab)**	SLL	**(L-Dab)**	TLS	**(L-Dab)**	A**K**AA**K**L	**(L-Dab)**	TLL	**(L-Dab)**	ALS	**(L-Dab)**	-amide
**D105 (Lys^1^-6 Dap-1)**	**+9**	Ac-KL	**(L-Dap)**	SLL	**(L-Dap)**	TLS	**(L-Dap)**	A**K**AA**K**L	**(L-Dap)**	TLL	**(L-Dap)**	ALS	**(L-Dap)**	-amide
**D101 (Lys^1^Ser^26^-5 Lys-1)**	**+8**	Ac-KL	**(Lys)**	SLL	**(Lys)**	TLS	**(Lys)**	A**K**AA**K**L	**(Lys)**	TLL	**(Lys)**	ALS	**(Ser)**	-amide
**D102 (Lys^1^Ser^26^-5 Dab-1)**	**+8**	Ac-KL	**(L-Dab)**	SLL	**(L-Dab)**	TLS	**(L-Dab)**	A**K**AA**K**L	**(L-Dab)**	TLL	**(L-Dab)**	ALS	**(Ser)**	-amide
		1	3		7			14	15		22		26	
		KL	**X**	SLL	**X**	TLSAA K		**X**	**X**	**K**LATLL	**X**	ALS	**X**	
**D88 (Lys^1^-6 Lys-2)**	**+9**	Ac-KL	**(Lys)**	SLL	**(Lys)**	TLSAA **K**		**(Lys)**	**(Lys)**	**K**LATLL	**(Lys)**	ALS	**(Lys)**	-amide
**D89 (Lys^1^-6 Dab-2)**	**+9**	Ac-KL	**(L-Dab)**	SLL	**(L-Dab)**	TLSAA **K**		**(L-Dab)**	**(L-Dab)**	**K**LATLL	**(L-Dab)**	ALS	**(L-Dab)**	-amide
**D106 (Lys^1^-6 Dap-2)**	**+9**	Ac-KL	**(L-Dap)**	SLL	**(L-Dap)**	TLSAA **K**		**(L-Dap)**	**(L-Dap)**	**K**LATLL	**(L-Dap)**	ALS	**(L-Dap)**	-amide
**D103 (Lys^1^Ser^26^-5 Lys-2)**	**+8**	Ac-KL	**(Lys)**	SLL	**(Lys)**	TLSAA **K**		**(Lys)**	**(Lys)**	**K**LATLL	**(Lys)**	ALS	**(Ser)**	-amide
**D104 (Lys^1^Ser^26^-5 Dab-2)**	**+8**	Ac-KL	**(L-Dab)**	SLL	**(L-Dab)**	TLSAA **K**		**(L-Dab)**	**(L-Dab)**	**K**LATLL	**(L-Dab)**	ALS	**(Ser)**	-amide

a The D denotes that all amino acid residues in each peptide are in the D-conformation except for L-Dab and L-Dap residues which are in the L conformation. Specificity determinants are positively charged residues in the center of the non-polar face (Lys13/Lys16) (Figure 2).

b Peptide sequences are shown using the one-letter code for all amino acid residues except at positions X, where the three-letter code is used. Ac denotes N^α^-acetyl and amide denotes C^α^-amide. Positions X are positively charged residues (Lys, L-Dab and L-Dap) on the polar face of the amphipathic α-helix (Figure 1); −1 denotes 6 positively charged residues on the polar face at positions 3, 7, 11, 18, 22 and 26 or 5 positively charged residues on the polar face at positions 3, 7, 11, 18 and 22 (position 26 is substituted by Ser); −2 denotes 6 positively charged residues on the polar face at positions 3,7, 14, 15, 22 and 26 or 5 positively charged residues on the polar face at positions 3, 7, 14, 15 and 22 (position 26 is substituted by Ser).

**Table 2:** Antibacterial activity against 7 strains of *Acinetobacter baumannii* resistant to Polymyxin B and Colistin, hemolytic activity and therapeutic index

Peptide Name^a^	Peptide Mass	MIC(μm)^b^							MIC_GM_ (μM)^b^	HC_50_(μM)^c^	T.I.^d^
		MB9941	MB9949	MB9951	MB9952	MB9953	MB9955	MB9963			
**D84(Lys^1^-6 Lys-1)**	2865.6	0.3	0.7	0.3	0.7	0.7	0.3	0.7	0.5	54.3	108.6
**D86(Lys^1^-6 Dab-1)**	2697.3	1.5	0.8	0.8	0.8	0.8	0.8	0.8	0.9	>742	>824
**D105(Lys^1^-6 Dap-1)**	2613.1	0.8	0.8	3	0.8	3	0.5	1.5	1.2	>1148	>957
**D101(Lys^1^Ser^26^-5 Lys-1)**	2824.5	0.7	0.7	0.7	0.7	1.5	0.7	0.7	0.8	103.9	129.9
**D102(Lys^1^Ser^26^-5 Dab-1)**	2684.3	0.4	0.7	0.7	0.7	1.4	0.7	0.7	0.7	>708	>1012
**D88(Lys^1^-6 Lys-2)**	2865.6	0.7	0.7	0.3	0.3	0.3	0.3	0.4	0.4	80.6	201.5
**D89(Lys^1^-6 Dab-2)**	2697.3	0.7	0.7	0.7	0.7	0.7	0.7	0.7	0.7	>1112	>1589
**D106(Lys^1^-6 Dap-2)**	2613.1	0.8	0.8	1.5	0.8	0.8	0.4	0.8	0.8	340.2	425.3
**D103(Lys^1^Ser^26^-5 Lys-2)**	2824.5	0.7	0.7	0.7	0.7	0.7	0.7	0.7	0.7	134.9	192.7
D104(Lys^1^Ser^26^-5 Dab-2)	2684.3	0.7	0.7	0.7	0.7	1.5	0.7	0.7	0.8	>1490	>1863
**Colistin**	1155.5	>28	>28	>28	>28	>28	>28	>28	>28		
**Polymyxin B**	1301.6	>25	>25	>25	>25	>25	>25	>25	>25		

a The sequences and the −1 or −2 designations are described in table 1.

b MIC is minimal inhibitory concentration (μM) that inhibited growth of different strains in Mueller-Hinton (MH) medium at 37°C after 24h, with the MIC based on three sets of determinations; MIC_GM_ is the geometric mean of the MIC values from seven different strains of *Acinetobacter baumanii* resistant to Polymyxin B and Colistin, antibiotics of last resort. Colistin and Polymyxin B results provided by MERCK.

c Hemolytic activity, HC_50_, is the concentration of peptide that results in 50% hemolysis of human red blood cells after 18 h at 37°C.

d Therapeutic index (T.I.) was calculated from HC_50_ (μM)/MIC_GM_ (μM).

**Table 3:** Biophysical data.

Peptide Name^a^	Net charge	Hydrophobicity^b^	Aqueous pH 7		50% TFE	Δ[θ]_222_	% Helix^d^ Induced
		pH 2 t_R_	[θ]_222_^c^	% Helix^d^	[θ]_222_^c^	TFE-aqueous	
**With specificity determinants**							
**D84(Lys^1^-6 Lys-1)**	+9	136.9	8,230	24	33,653	25,423	76
**D86(Lys^1^-6 Dab-1)**	+9	113.9	1,769	6	27,923	26,154	94
**D105(Lys^1^-6 Dap-1)**	+9	126.6	5,961	25	24,192	18,231	75
**D101(Lys^1^Ser^26^-5 Lys-1)**	+8	139.4	2,538	7	38,538	36,000	93
**D102(Lys^1^Ser^26^-5 Dab-1)**	+8	112.2	5,962	25	23,385	17,423	75
**D88(Lys^1^-6 Lys-2)**	+9	135.5	9,154	28	33,115	23,961	72
**D89(Lys^1^-6 Dab-2)**	+9	115.8	1,692	6	27,923	26,231	94
**D106(Lys^1^-6 Dap-2)**	+9	128.8	4,462	19	23,231	18,769	81
**D103(Lys^1^Ser^26^-5 Lys-2)**	+8	137.9	8,462	29	29,114	20,692	71
**D104(Lys^1^Ser^26^-5 Dab-2)**	+8	115.2	1,462	6	23,000	21,538	94

a The D denotes that all amino acids in each peptide are in the D-conformation except where noted table 1.

b t_R_ denotes retention time in RP-HPLC at pH 2 at a temperature of 25°C, and is a measure of overall peptide hydrophobicity.

c The mean residue molar ellipticities [θ]_222_ (mdeg cm^2^/(dmol*res)) at a wavelength of 222 nm were measured at 25°C in aqueous conditions (100 mM KCl, 50 mM Na_2_HPO_4_/NaH_2_PO_4_, pH 7.0) or in aqueous buffer containing 50% trifluoroethanol (TFE) by circular dichroism spectroscopy.

d The helical content (as a percentage) of a peptide is relative to the molar ellipticity value of the peptide in the presence of 50% TFE. % helix induced is the increase in molar ellipticity (as a percentage) of the peptide in the presence of 50% TFE.

## Abbreviations

AMPs: Antimicrobial Peptides
Dab: Diaminobutyric Acid
Dap: Diaminopropionic Acid
HC_50_: Peptide concentration that results in 50% lysis of human red blood cells (hemolytic activity)
MIC_GM_: Minimal inhibitory concentration geometric mean of the number of strains tested
T.I: Therapeutic Index
TFE: 2,2,2-Trifluoroethanol
